# Development and validation of an automated emergency department-based syndromic surveillance system to enhance public health surveillance in Yukon: a lower-resourced and remote setting

**DOI:** 10.1186/s12889-021-11132-w

**Published:** 2021-06-29

**Authors:** Etran Bouchouar, Benjamin M. Hetman, Brendan Hanley

**Affiliations:** 1 Department of Health and Social Services, Government of Yukon, Whitehorse, Canada; 2 College of Public Health, University of South Florida, Tampa, FL USA; 3 Canadian Field Epidemiology Program, Public Health Agency of Canada, Ottawa, ON Canada; 4 Department of Population Medicine, University of Guelph, Guelph, ON Canada

**Keywords:** Syndromic surveillance, Mass gathering, Detection algorithm, COVID-19, Case definitions

## Abstract

**Background:**

Automated Emergency Department syndromic surveillance systems (ED-SyS) are useful tools in routine surveillance activities and during mass gathering events to rapidly detect public health threats. To improve the existing surveillance infrastructure in a lower-resourced rural/remote setting and enhance monitoring during an upcoming mass gathering event, an automated low-cost and low-resources ED-SyS was developed and validated in Yukon, Canada.

**Methods:**

Syndromes of interest were identified in consultation with the local public health authorities. For each syndrome, case definitions were developed using published resources and expert elicitation. Natural language processing algorithms were then written using Stata LP 15.1 (Texas, USA) to detect syndromic cases from three different fields (e.g., triage notes; chief complaint; discharge diagnosis), comprising of free-text and standardized codes. Validation was conducted using data from 19,082 visits between October 1, 2018 to April 30, 2019. The National Ambulatory Care Reporting System (NACRS) records were used as a reference for the inclusion of International Classification of Disease, 10th edition (ICD-10) diagnosis codes. The automatic identification of cases was then manually validated by two raters and results were used to calculate positive predicted values for each syndrome and identify improvements to the detection algorithms.

**Results:**

A daily secure file transfer of Yukon’s Meditech ED-Tracker system data and an aberration detection plan was set up. A total of six syndromes were originally identified for the syndromic surveillance system (e.g., Gastrointestinal, Influenza-like-Illness, Mumps, Neurological Infections, Rash, Respiratory), with an additional syndrome added to assist in detecting potential cases of COVID-19. The positive predictive value for the automated detection of each syndrome ranged from 48.8–89.5% to 62.5–94.1% after implementing improvements identified during validation. As expected, no records were flagged for COVID-19 from our validation dataset.

**Conclusions:**

The development and validation of automated ED-SyS in lower-resourced settings can be achieved without sophisticated platforms, intensive resources, time or costs. Validation is an important step for measuring the accuracy of syndromic surveillance, and ensuring it performs adequately in a local context. The use of three different fields and integration of both free-text and structured fields improved case detection**.**

**Supplementary Information:**

The online version contains supplementary material available at 10.1186/s12889-021-11132-w.

## Introduction

Emergency department-based syndromic surveillance systems (ED-SyS) are effective tools that complement laboratory-based surveillance methods for the detection of infectious diseases and other public health aberrations, and enable effective control measures in a timely response [1–4]. These ED-SyS automate the use of existing (near-) real-time pre-diagnostic data routinely collected in hospitals and apply statistical algorithms to detect aberrations for immediate public health investigation. ED-SyS are routinely used in urban locations to monitor diseases of public health importance and have been leveraged globally during mass gathering events [5–8]. Many of these systems depend on (1) dedicated technical human resources (e.g., epidemiologists and data scientists), (2) sophisticated technological IT platforms (e.g., ESSENCE, BioSense, and NC-Detect in the U.S.; PHIDO and ACES in Canada) [9–12], and the use of (3) resource intensive syndrome case validation methods [1, 13]. Often, these resources and capacities are limited or unavailable in health departments serving rural and remote communities [14]. There is a need to identify methods that lower-resourced communities can use to develop and operate automated ED-SyS that approach the efficacy of a fully validated real-time system, without the steep setup time and resource costs required by more sophisticated platforms. This paper describes a novel, near real-time automated ED-SyS that was developed and validated using minimal resources in preparation of a mass gathering event in Yukon, a low resourced northern territory in Canada.

Yukon has a population of approximately 41,761 people with the majority (78%) living in the capital city of Whitehorse and 22% living in smaller remote communities [15]. Yukon’s Department of Health and Social Services (HSS) is responsible for the delivery of public health services and faces similar resource constraints to those experienced by rural local health departments in the U.S. including limited human resources (i.e., fewer staff, staff turnover, and a lack of specialty positions such as epidemiologists), information technology, and funding [14]. Yukon’s Department of HSS only recently created its first epidemiologist position, many program areas do not have the skills or capacity to manage data, IT supports are limited, and it is common for a single staff to be responsible for a wide range of acute care and public health functions. These are all factors taken into consideration while planning for enhanced surveillance of infectious diseases ahead of the Arctic Winter Games (AWG), a biennial international sporting and cultural event that rotates between major centers in the circumpolar North. In other mass-gathering sporting events, vaccine-preventable diseases such as measles, influenza, and meningococcal and gastrointestinal illnesses have been reported [7, 16].

Past gatherings for the Arctic Winter Games in Whitehorse have seen the use of syndromic surveillance systems successfully applied, with reports hand-collected from field medical staff, polyclinics, and the hospital emergency department. These data were then manually entered into a spreadsheet-based software and assessed using a cumulative-sum based approach to identify trends higher than anticipated during a short window of time [17]. It is generally accepted that investment in mass gathering surveillance should provide infrastructure that is sustainable for long-term use [7], however, this historically has not been the case in Yukon. While effective for short-term surge support, the resources required to run previous syndromic surveillance systems (e.g., refocusing full-time staff, mobilized federal field epidemiologists) were too steep to be maintained. To leverage the benefits provided by syndromic surveillance in the Yukon permanently, proposed tools must be simple to implement and train staff to use while requiring limited human and financial resources.

Similarly, to properly support an ED-SyS for long-term implementation, operational case definitions must be developed and validated. These algorithms influence the balance between identifying all possible cases (i.e., sensitivity) and excluding those without the disease of interest (i.e., specificity) [4, 18, 19]. There is no gold standard approach for developing or validating ED-SyS case definitions; methods described in the literature suggest developing definitions using expert-based consensus followed by an ongoing refinement process, with validation based on chart review by more than one clinician or comparison with a gold-standard dataset [18–26]. Most case definitions rely on natural language processing algorithms that identify keywords associated with a syndrome of interest in the chief complaint (CC) field and discharge diagnosis (DD) fields [3, 25, 27–33], with the use of clinical triage notes (CN) emerging more recently [25, 34–36]. Several studies have noted disagreement between syndrome definitions when using CC fields versus DD fields but, in general, case detection improves when leveraging information from both fields [20, 25, 32, 34, 35, 37, 38]. The use of CN fields also increases the sensitivity of the definition but may decrease specificity [25, 34–36].

Analyzing unstructured free-text commonly contained in these fields is not without its set of challenges, where spelling, abbreviations, and negating vocabulary issues can increase the risk of false-positives [25, 34, 36]. On the same note, case detection can be further limited by challenges in identifying all relevant syndrome terms or new terms introduced in documentation [38]. The lack of standardized codes can lead to variations in documentation between jurisdictions and/or facilities where application of one syndrome case definition may not fare as well in other without considering the local context [38]. While structured standardized codes can overcome many of these challenges, they may face sensitivity and specificity limitations [39]. Outside ICD diagnosis codes, the literature on the use of structured CC fields such as the use of CEDIS codes is limited.

The objectives of the present study are to describe the development and validation of Yukon’s first automated ED-SyS and evaluate the effectiveness of three different ED data fields. By measuring the contribution different sources of free-text (e.g., CN, and DD fields) with standardized codes (e.g., CEDIS), we aim to identify an optimal set of natural language processing algorithms to be used as part of a local ED-SyS to be implemented for surge support as well as lasting capacity in a resource-constrained setting where staff and technological infrastructure are limited.

## Methods

The design and validation of Yukon’s ED-SyS occurred between November 2019 and February 2020, involving the following stages: (1) initial review of available data sources; (2) development of initial case definitions; (3) development of natural language processing algorithms and logic; (4) validation of ED-SyS using validation dataset; (5) refinements to algorithms and logic.

### Data source

The Territorial Epidemiologist collaborated with health information analysts at Yukon’s Whitehorse General Hospital (WGH), the only hospital in Whitehorse and Yukon’s primary hospital to access records from the newly implemented real-time emergency department electronic records collection tool: Meditech ED-Tracker system. This system comprises of an electronic medical records database that captures the following information at the time of patient assessment (1) demographic characteristics and date of visit (2) clinical notes (CN) (e.g., free-text describing a brief history of the stated complaint, the recorded temperature (in °C) at triage) (3) the chief complaint (CC) containing the Canadian Emergency Department Information System (CEDIS) code [40, 41] and (4) the physician discharge diagnosis (DD) which is a free-text field providing the diagnosis at patient discharge from the ED. Collaboration between the territorial epidemiologist and WGH stakeholders (the custodians of the data) and the completion of a privacy impact assessment (PIA) led to the establishment of a daily (1 day lag) secure file transfer of ED visit data to the Office of the Chief Medical Officer of Health (OCMOH) in November 2019. Historical data of all ED visits from January 1, 2018 onwards were also made available for use. However, data between January 1, 2018 and September 30, 2018 were deemed inconsistent (e.g., incomplete data and/or missing fields) and therefore excluded from further use. National Ambulatory Care Reporting System (NACRS) records were used as a reference in our validation process for the inclusion of International Classification of Disease, 10th edition (ICD-10) codes; NACRS data were thus extracted for the time period of October 1, 2018 to April 30, 2019 and merged with ED-Tracker data. Observations without a match between the two datasets were dropped. All analyses were performed using Stata LP 15.1 (Texas, USA).

### Development of initial case definitions

Syndromes of interest were identified via consensus between Yukon’s Communicable Disease Control program (YCDC) manager, the office of the Chief Medical Officer of Health (OCMOH), and the territorial epidemiologist. A review of existing syndromic surveillance platforms (e.g., NC-Detect, ESSENCE-II) [42, 43] along with current literature were used to inform the terms used in the initial case definitions for each syndrome [3, 13, 18, 21, 22, 24–29, 31, 32, 34, 44–50]. These sources enabled us to create an initial list of key terms that have been used for monitoring of similar syndromes of interest. Terms were then further refined for local context by review from stakeholders at YCDC and the OCMOH. For example, in clinical triage notes, the nurse attendant may capture inebriation or alcohol abuse by using the short-hand term ‘EtOH’; several examples such as this were included in our NLP algorithms to ensure adequate capture of these types of locally adapted data collection terms. In addition to key terms, CEDIS codes related to each syndrome were also identified and included in case definitions. Additionally, a syndrome related to COVID-19 was developed following recommendations published by the World Health Organization (WHO) [51] and local consensus with the OCMOH. This case-finding definition was further developed through extensive internal review, a review of WHO’s COVID-19 weekly Situation Reports [52], and recommendations made available online by the Public Health Agency of Canada [53].

For each syndrome, individual terms and codes that were identified through literature review or expert consensus were organized under more broadly defined concepts that were then used to determine whether a patient record met the case definition for the syndrome of interest (see Supplement A1). Using these key terms and concepts we developed natural language processing algorithms in Stata and applied these to query ED-Tracker records from October 1, 2018 to April 30, 2019 as this dataset coincided with available NACRS data that could be used to for downstream validation purposes. Algorithms were constructed using regular expressions (i.e., the “*regexm*” function in Stata) to detect full or partial strings from the list of key terms and concepts in either the CN, CC, or DD fields. In general, algorithms were applied for each syndrome to query data fields using a forward-inclusion strategy, starting with DD, then querying the CC and CN fields to determine whether or not a case record met the case definition for any of the syndromes of interest (see Table 1).

**Table 1:** Case definitions and algorithm logic for syndromes of interest in Yukon’s ED-SyS

Syndrome	Definition	Logic/Algorithm	Disease/condition of Interest
**Gastrointestinal (GI)**	Match with the following diagnosis of interest **OR** Diarrhea Concept **OR** Vomiting/Nausea Concept **OR** Nausea Only Concept Anorexia Concept **OR** GI Concept GI related EXCLUSIONS TERMS	1. Flag DD logic first if not blank or left without being seen related text 2. If logic step #1 not met and CN/CC logic =1 then flag 3. If DD = “Viral” and DD =0 and CN/CC logic = 1 then flag 4. If DD logic met and CN/CC logic met then then flag	Verotoxigenic *E. coli* (e.g. O157:H7) enteric disease; Campylobacteriosis; Cryptosporidiosis; Salmonellosis; Shigellosis; Yersiniosis; Gastroenteritis (acute diarrheal)/ Enteric outbreaks all causes
**Influenza-Like Illness (ILI)**	Match with the following diagnosis of interest **OR** ILI **OR** Fever Concept **AND** Cough Concept **AND** (sore throat Concept **OR** at least one constitutional sign/symptom: Anorexia Concept **OR** Dizziness Concept **OR** Malaise Concept **OR** Muscle Pain Concept **OR** Lymph Concept)	1. If DD logic or CN/CC logic met then flag	Influenza
**Respiratory (Resp)**	Match with the following diagnosis of interest **OR** Respiratory Concept **OR** Cough Concept **OR** Sore Concept **AND** (at least one constitutional sign/symptom: Anorexia Concept **OR** Dizziness Concept **OR** Fever Concept **OR** Malaise Concept **OR** Muscle Pain Concept **OR** Lymph Concept)	1. Flag DD logic first if not blank or left without being seen related text 2. If logic step #1 not met and CN/CC =1 then flag 3. If DD logic met and CN/CC logic met then then flag	Pertussis (whooping cough), Influenza, RSV
**Rash**	Match with the following diagnosis of interest **OR** Rash Concept **AND** at least one constitutional sign/symptom: (Anorexia term **OR** Dizziness Concept **OR** Fever Concept **OR** Malaise Concept **OR** Muscle Pain Concept **OR** Lymph Concept) Rash related EXCLUSION terms	1. If DD logic or CN/CC logic met then flag	Measles, Rubella, Chickenpox, Meningitis, Scarlet Fever
**Mumps**	Match with the following diagnosis of interest **OR** parotitis **OR** orchitis **OR** Swelling Concept **AND** Face Concept Mumps related EXCLUSIONS	1. Flag DD logic first if not blank or left without being seen related text 2. If logic step #1 not met and CN =1 then flag 3. If DD logic met and CN logic met then then flag	Mumps
**Neurological infections (Neuro)**	Match with the following diagnosis of interest **OR** Fever Concept **AND** (stiff neck Concept **OR** Seizures Concept **OR** Blurred Vision Concept **OR** Photophobia Concept **OR** Altered Mental Status Concept **OR** Headache Concept **OR** Paralysis Concept)	1. Flag DD logic first if not blank or left without being seen related text 2. If logic step #1 not met and CN/CC logic =1 then flag 3. If DD = “Viral”|"Febrile seizure”|"Headache nyd”|"Malaise nyd”|"Sepsis” and DD logic =0 and CN/CC logic = 1 then flag 4. If DD logic met and CN/CC logic met then then flag	Invasive meningococcal disease (Neisseria meningitis) infection; Encephalitis/ meningitis (bacterial; parasitic; viral)
**Coronavirus Disease 2019 (COVID-19)**	ED visits prior to April 16 *takes into consideration negating terms Match with the diagnosis of interest in DD OR CC (SOB, Cough, Fever) on or after Jan 1, 2020 AND Match with any of the following: -countries listed from WHO Sitrep to have “local transmission”-indicates locations where the source of infection is within the reporting location Jan 1, 2020 to March 19, 2020 -Mention of international or travel in CN on or after March 15, 2020 OR -Match with the diagnosis of interest (COVID) in CN ED visits on or after April 16 *takes into consideration negating terms -Match with the diagnosis of interest in DD OR -Match with the diagnosis of interest in CN OR -Match with any of the following: -CC on or after Jan 1, 2020- SOB, Cough, Fever -CC on or after April 16, 2020- Chestpnonc, Stridor, Wheezing, Sore Throat, Syncope, Genweak, Headache, URTI, Nasal congestion/hayfever, Altered LOC, Confusion, Sensory loss/parathesis, Rep Arrest, Cyanosis -CN- “Loss of taste” or “Loss of smell” AND Match with any of the following: -countries listed from WHO Sitrep to have local transmission-indicates locations where the source of infection is within the reporting location Jan 1, 2020 to March 19, 2020 -Mention of international or travel in CN on or after March 15, 2020		COVID-19

### Validation and refinement of initial syndromic surveillance case definitions

Records with an exact match between ED-Tracker and NACRS datasets (i.e., visits occurring between October 1st, 2018 and April 30th, 2019) were used to validate the ED-SyS. Note: algorithms for detecting gastrointestinal and respiratory syndromes resulted in what was pragmatically considered too many records for manual review with current staff resources (e.g., > 600 records per syndrome), therefore an abbreviated dataset containing records from November 1, 2018 to January 31, 2019 was used instead for validating these two syndrome groups. Following the example set by [24], records flagged for meeting syndromic case definitions were assigned one of three validation classifications: likely related to the disease of interest (true-positive) = “0”; unclear/conflicting information (false positive) = “1”; does not match case definition/not related (false positive) = “2” (Table 1). These assignments were carried out first by reviewing available ICD-10 codes; any flagged case with an ICD-10 code equivalent to the those outlined in [54] was assigned a zero (“0”). This step was accomplished using the US-CDC’s database of equivalent code translation [55]. Remaining records were validated manually by two epidemiologists reviewing the ICD-10 code, DD, CC and CN fields, typically in that order. After an initial review, a random sample of 10 cases was taken from the “0”, “1”, and “2” sub-groups for each syndrome (30 cases total per syndrome) and reviewed by both epidemiologists to ensure agreement and consistency among classification. Discrepancies in scoring were discussed, and corrections were retroactively applied to the validated datasets so that consensus for all scores was achieved.

Following validation, we manually reviewed the validated records from each syndrome to identify: (1) misspellings or shorthand among the free-text fields that were not considered during the development of the initial algorithms; (2) terms and CEDIS codes that could be used to further refine the inclusion or exclusion of true- and false-positives, respectively; and (3) adjustments to the algorithm structure to improve case classification.

### Evaluating the performance of the ED-SyS

Each syndrome included in the ED-SyS was evaluated by measuring the positive predictive value (PPV) using the validated case records. The PPV was defined as the total number of true-positive records (classification = “0”) over the total number of records (classification = “0”, “1”, or “2”), as a percent. To evaluate the contribution of each data field on the overall performance of the ED-SyS, we measured the PPV from each component individually as well as in combination (e.g., CN; CC; DD; CN + CC; CN + CC + DD). Finally, to evaluate which terms were most influential in identifying each syndrome, the frequency of terms present among true-positive case records were measured and visually assessed using word clouds (NB: select terms for the construction of the word clouds were double-counted as some concept terms exist in both CN and DD algorithms).

## Results

### Description of Yukon’s ED-SyS

**Fig. 1:**
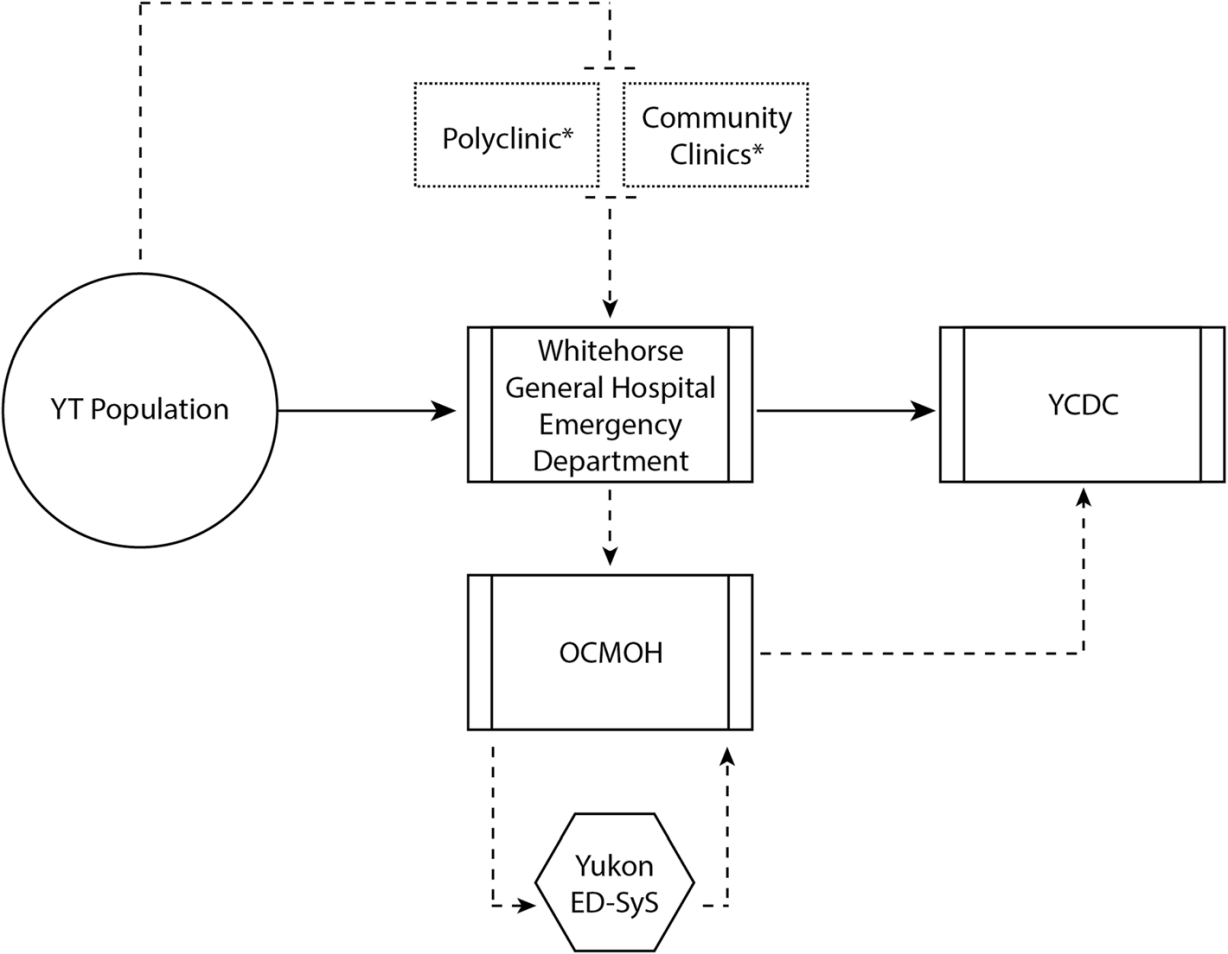
An overview of Yukon’s ED-SyS. Visit records from the Yukon population is entered into the ED-TRACKER system via Whitehorse General Hospital’s ED staff upon visit to the hospital. Data from this database are automatically downloaded daily by the OCMOH, where the territorial epidemiologist queries the dataset using the ED-SyS to identify syndromes of interest that may be flagging. When aberrations are detected that require follow-up, the OCMOH notifies YCDC to activate further investigation. Dashed boxes marked by an asterix (*) indicate modular components of the system that are designed to be used in a temporary surge capacity during mass-gathering events

An overview of the Yukon ED-SyS is presented in Fig. 1. In Yukon, the general Whitehorse population can access emergency and primary care services through the WGH-ED, with primary care services also accessible through community clinics. These are both access points where symptomatic individuals with infectious diseases of interest present for clinical assessment. Positive results from the WGH hospital Laboratory serve as the territory’s laboratory-based surveillance system for reportable infectious diseases. The ED-SyS described here complements the laboratory component of the system, providing a source for pre-diagnosis data in Yukon sent electronically to the OCMOH daily for analysis. Once an extract of ED-SyS data is received at the OCMOH, the Territorial Epidemiologist runs the NLP syndrome algorithm and inputs the syndrome aggregate counts into the US CDC’s Early Aberration Reporting System (EARS-X) to detect any statistically significant increases in counts that may warrant further investigation. This adaptive control chart detection tool uses three algorithms (C1, C2, and C2) based on a positive 1-sided CUSUM calculation to detect aberrant data. The C1 algorithm generates an alert if counts are 3 standard deviations above the moving 7-day baseline, C2 incorporates an additional 2-day window between the observed count and baseline, and C3 uses the sum of C2 and the previous 2 days [11, 56]. EARS-X was selected for use as it required minimal historical baselines, minimal training for use, and was readily accessible. Alerts generated are then reviewed by the Territorial Epidemiologist for any evidence of clustering by age, geography, gender, chief complaint, and discharge diagnosis. Evidence of clustering are forwarded along to YCDC for clinical assessment resulting in one of the following: (1) an investigation, (2) continued monitoring, or (3) no action.

In total, seven syndromes of interest were selected for daily monitoring: gastrointestinal illness (GI), influenza-like-illness (ILI), mumps, neurological infections (Neuro), rash, respiratory illnesses (Resp), and COVID-19. For each syndrome, diseases/conditions of interest were identified, and case definitions were developed based on algorithms that query information from the WGH ED-Tracker database (see Table 1). A complete list of terms used to query each syndrome is available in supplemental material (Supplement A1).

### ED-SyS validation and performance

The merged dataset of ED visit data from the ED Tracker and NACRS contained a total of 19,023 unique ED visits between October 1, 2018 and April 30, 2019. After applying the initial case definitions to this dataset, hits to the GI and Resp syndromes greatly exceeded our practical ability to manually validate flagged records (e.g., over 1000 results per syndrome). For these two syndromes, a subset of ED visits from November 1, 2018 to January 31, 2019 was used for validation (*n* = 8246 records). After adjustment, our case definitions flagged 3707 ED visits as potential cases for all syndromes (1793 for GI; 966 for Resp; 593 for ILI; 64 for Rash; 234 for Neuro; 57 for Mumps; and 0 for COVID-19). As no records were flagged for COVID-19, the syndrome was not included in validation procedures.

Results from our validation are presented in Table 2. Among our initial case definitions, DD consistently returned the highest proportion of true positive cases (PPV: 51.3 to 100%), compared to CN (PPV: 22.8 to 86.1%) and CC (PPV: 0 to 35.0%) when used individually. In general, the CN field produced the most sensitive results, flagging the highest number of visits for all syndromes, while CC and DD fields provided a lower number of hits with higher specificity. These trends were maintained after adjustment to the initial case definitions, with the largest improvements observed in the GI syndrome (e.g., PPV for DD improved from 51.3 to 86.9%).

**Table 2:** Flagged cases for each syndrome from Whitehorse General Hospital ED-Tracker records, Oct. 1, 2018 – Apr. 30, 2019

Flagged visits by separate fields					Flagged visits by combined fields				
Field	# Hits	PPV (CI)	# Hits	PPV (CI)	Field(s)	# Hits	PPV (CI)	# Hits	PPV (CI)
**Gastrointestinal**					**Gastrointestinal**				
Pre-validation			Post-Validation		Pre-validation			Post-Validation	
CN	1579	0.23 (0.18–0.27)	746	0.44 (0.38–0.49)	CN	1579	0.23 (0.18–0.27)	746	0.44 (0.38–0.49)
CC	866	0.35 (0.30–0.40)	295	0.40 (0.31–0.48)	CN/CC	1644	0.22 (0.18–0.26)	759	0.45 (0.39–0.50)
DD	485	0.51 (0.45–0.57)	236	0.87 (0.82–0.91)	CN/CC/DD	601	0.49 (0.43–0.54)	316	0.79 (0.74–0.84)
**Respiratory**					**Respiratory**				
Pre-validation			Post-Validation		Pre-validation			Post-Validation	
CN	639	0.75 (0.71–0.78)	625	0.75 (0.71–0.78)	CN	639	0.75 (0.71–0.78)	625	0.75 (0.71–0.78)
CC	19	0.21 (0–0.59)	19	0.21 (−0.19–0.59)	CN/CC	662	0.74 (0.70–0.77)	647	0.74 (0.70–0.77)
DD	76	0.92 (0.86–0.98)	76	0.92 (0.86–0.98)	CN/CC/DD	548	0.84 (0.81–0.87)	542	0.84 (0.81–0.87)
**ILI**					**ILI**				
Pre-validation			Post-Validation		Pre-validation			Post-Validation	
CN	361	0.86 (0.82–0.90)	356	0.87 (0.83–0.91)	CN	361	0.86 (0.82–0.90)	356	0.87 (0.83–0.91)
CC	0	–	0	–	CN/CC	380	0.85 (0.81–0.89)	375	0.86 (0.82–0.90)
DD	141	0.96 (0.92–0.99)	141	0.96 (0.92–0.99)	CN/CC/DD	482	0.87 (0.84–0.90)	477	0.88 (0.85–0.91)
**Rash**					**Rash**				
Pre-validation			Post-Validation		Pre-validation			Post-Validation	
CN	9	0.56 0.12–0.97)	9	0.56 (0.12–0.97)	CN	9	0.56 (0.12–0.97)	5	0.56 (0.12–0.97)
CC	0	–	0	0 (0)	CN/CC	56	0.57 (0.40–0.73)	56	0.57 (0.40–0.73)
DD	10	0.90 (0.70–1.00)	10	0.90 (0.70–1.09)	CN/CC/DD	64	0.63 (0.47–0.77)	64	0.63 (0.47–0.77)
**Neurological**					**Neurological**				
Pre-validation			Post-Validation		Pre-validation			Post-Validation	
CN	201	0.35 (0.24–0.45)	193	0.35 (0.24–0.46)	CN	201	0.35 (0.24–0.45)	193	0.35 (0.24–0.46)
CC	0	–	0	–	CN/CC	167	0.54 (0.44–0.64)	160	0.56 (0.45–0.65)
DD	2	1.00 (1.00–1.00)	2	1.00 (1.00–1.00)	CN/CC/DD	6	0.83 (0.51–1.00)	19	0.89 (0.75–1.03)
**Mumps**					**Mumps**				
Pre-validation			Post-Validation		Pre-validation			Post-Validation	
CN	43	0.37 (0.14–0.60)	40	0.40 (0.16–0.63)	CN	43	0.37 (0.14–0.60)	40	0.40 (0.16–0.63)
DD	17	0.94 (0.83–1.00)	17	0.94 (0.83–1.05)	CN/DD	57	0.51 (0.33–0.68)	17	0.94 (0.83–1.05)

*Abbreviations*: *CN* clinical notes; *CC* chief complaint; DD discharge diagnosis; *PPV* positive predictive value; *CI* confidence interval calculated using a 95% confidence level

Data Source: Meditech ED Tracker

Note: Subject who meet case definition were those whose ED visit was assessed to be related to the disease/condition of interest. Combined field may include OR statements and/or AND statements using information in the CN, CC, DD field. Post-validation results refer to application of NLP algorithms after improvements to the algorithms were made during the initial validation phase

Combining multiple levels of data from ED-Tracker produced an average of 6.7-fold more hits to each syndrome than querying each component individually (Table 2). The largest example of this was observed when querying for visits related to the Resp Syndrome; using CC alongside CN increased hits from our original case definitions over 33-fold. Using our final case definitions for the combined fields produced the largest improvements to the GI Syndrome, with the PPV for the CN/CC combination increasing to 44.5% from 22.3% and the CN/CC/DD combination increasing to 78.8% from 48.8%. Changes observed among the other syndromes were minimal, save for the PPV of detecting the Mumps Syndrome, which increased from 50.9 to 94.1%, although the total number of observed hits decreased from 57 to 17.

### Evaluating terms and logic used to identify syndromes

After an initial review of the ED-SyS performance using our validation dataset, several adjustments were made to the terms and algorithms used in our ED-SyS to improve the system’s performance within the local context. In general, three areas were useful in redefining ED-SyS queries to provide more accurate results in the Yukon setting: *(1) Misspellings or shorthand among the free-text fields that were not considered during the development of the initial algorithms.* For example, the acronym “LWBS” in the DD field was often used to indicate a patient had “left without being seen”, and additional acronyms were needed to describe the diarrhea, nausea, and vomit concepts within the GI syndrome case definition including “N&V” (nausea and vomiting), “N, V, D” (nausea, vomiting, diarrhea), and “V/D” (vomiting/diarrhea). *(2) Terms and CEDIS code that could be used to further refine the inclusion or exclusion of true- and false-positives, respectively*. For example, additional negating terms were identified for several syndromes; the inclusion of “no”, “denies” and “–” were therefore added to indicate negation within the diarrhea, nausea, vomit, fever, and cough concepts. Several CEDIS codes were consistently present among true- and false-positive results for each syndrome; we used these codes to help provide additional specificity to our inclusion and exclusion criteria based on local context (Table 3). For example, a high proportion of visits flagged by our GI case definition presented with abdominal pain (CEDIS 251), but were validated as unclear/conflicting information (*n* = 91/571, classification = “1”) or unrelated (*n* = 365/571, classification = “2”). For this reason, we elected to remove CEDIS 251 from the final GI syndrome case definition in addition to several related terms that were present in the original syndromic case definition at the CN and DD level (e.g., “ascites”, “LUQ”, “LLQ”, “RLQ”, “RUQ”, “diverticulitis”, “appendicitis”, “abdominal bloating”, and “flatus”) that were also associated with false-positive records. *(3) Adjustments to the algorithm logic that could improve the application of our algorithms*. For example, the original algorithm for detecting the Neuro syndrome queried the CN and CC fields only if the information from the DD field was blank. We modified this process to query CN and CC when the DD field contained at least one of the terms “febrile seizure,” “headache nyd,” “malaise nyd”, or “sepsis” without mention of the term “mening”. This improved both the sensitivity and specificity of the algorithm, increasing the number of hits from six to 19, while concomitantly increasing PPV from 83.3 to 89.5%.

**Table 3:** Positive Predictive Value (PPV) of selected CEDIS codes used to inform local inclusion or exclusion criteria for each syndrome using Whitehorse General Hospital ED-Tracker records from Oct. 1, 2018 – Apr. 30, 2019 (*n* = 19,023 records)

Syndrome and CEDIS code	Description	Hits (PPV%)	Inclusion / Exclusion
**Gastrointestinal** ^a^			
CEDIS-254	*“diarrhea”*	49 (22.8)	Inclusion
CEDIS-257	*“vomiting and/or nausea”*	170 (35.0)	Inclusion
CEDIS-852	*“fever”*	53 (51.3)	Inclusion
CEDIS-407	*“head injury”*	67 (0.0%)	Exclusion
CEDIS-307	*“UTI”*	30 (0.0%)	Exclusion
CEDIS-753	*“withdrawl”*	11 (0.0%)	Exclusion
CEDIS-251	*“abdominal pain”*	572 (20.1)	Exclusion
**Respiratory** ^a^			
CEDIS-653	*“cough”*	278 (85.6)	Inclusion
CEDIS-103	*“sorethroat”*	105 (84.8)	Inclusion
CEDIS-852	*“fever”*	103 (84.5)	Inclusion
**ILI**			
CEDIS-653	*“cough”*	284 (77.5)	Inclusion
CEDIS-103	*“sorethroat”*	97 (57.7)	Inclusion
CEDIS-852	*“fever”*	68 (92.6)	Inclusion
**Rash**			
CEDIS-708	*“rash”*	71 (73.2)	Inclusion
**Neurological**			
CEDIS-852	*“fever”*	33 (72.7)	Inclusion
CEDIS-404	*“headache”*	25 (68.0)	Inclusion
**Mumps**			
CEDIS-709	*“localized swelling/redness-abscess”*	17 (77.8)	Inclusion
CEDIS-104	*“neck swelling/pain”*	9 (73.2)	Inclusion
CEDIS-101	*“dental gum”*	3 (33.3)	Exclusion
CEDIS-102	*“facial trauma”*	3 (0.0)	Exclusion

*Abbreviations*: *CEDIS* Canadian Emergency Department Information System; *PPV* positive predictive value

^**a**^ These syndromes were applied to a subset of the total data containing 8246 records from November 1, 2018 to January 31, 2019

**Fig. 2:**
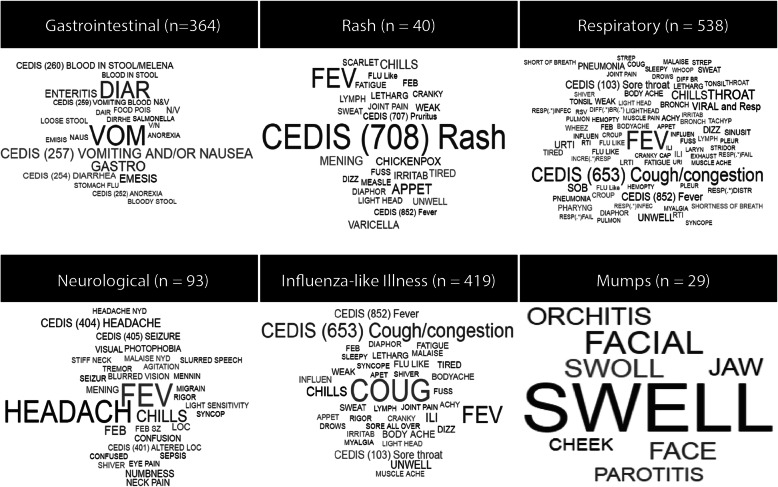
Word cloud of key terms and CEDIS codes found among true-positive validated case records. The size of each term is proportional to its frequency among validated case records

After completing the above adjustments to improve case-finding, we visualized the most influential terms among the true-positive case records for each syndrome (Fig. 2). Each syndrome appeared to have several terms that were essential to identifying the syndrome of interest. In three syndromes (Rash, Resp, and ILI) CEDIS codes appeared among the most frequent terms.

## Discussion

Our ED-SyS is the first automated system to be implemented in Yukon, and, to our knowledge in northern Canada. The advancements in data collection and management at the WGH made it possible to leverage this data source to develop an electronic ED-SyS for the territory. Partnerships between WGH, OCMOH and the Public Health Agency of Canada also greatly contributed to the timely design and implementation of the ED-SyS. The ED-SyS described here requires minimal resources to set up, can be adapted for local context, and does not require highly specialized or expensive software to run. Most importantly, the Yukon ED-SyS can be used for not only short-term surge capacity during mass gathering events but is useful as a permanent early-cluster detection system, improving the existing surveillance capacity for an otherwise low-resourced public health setting.

Results from our validation indicated that for each syndrome tested, free text from the CN field generally provided the most sensitive results, whereas the DD field was most specific. These results were not surprising to us, as the information stored in each field tended towards specificity of a diagnosis moving from CN to CC and DD and these findings were similar to those found in other studies [25, 34–36]. Our findings also highlight the value of leveraging the three fields together to improve case detection. This finding is noteworthy as a significant number of cases could be missed without considering one or more fields. This was particularly evident with our respiratory and ILI syndromes, where a significant number of cases could be missed without using the CN field. Other studies have noted improvements in case detection with combined fields. For example, one study noted that sensitivity improved for gastrointestinal, rash/fever, and ILI syndromes when CC, CN, and temperature fields were used together to more than 80% from 17 to 40% [36]. South et al. (2008) also improved sensitivity for detecting ILI up to 75% when combining multiple fields from the ED record (e.g., CC and CN) compared to sole chief complaint (27%) [26]. Similarly, another study also concluded that case detection for acute respiratory infections was improved by leveraging multiple fields (e.g., temperature, ICD-9 diagnosis code, treatment remedies field, and free text clinical notes) [34]. Results for the performance of individual fields towards the overall surveillance system should be interpreted with caution, however, as many of the algorithms were intended to pull information from CC and CN data only in the absence of a confirmed case or non-case from the DD field. For a more accurate assessment of the performance of each field, algorithms should be structured with the intent to use information uniquely from each data source, instead of in a combined fashion.

An important step in designing our ED-SyS was refining the system for local context with public health and emergency department nurses and collaborators operating the existing surveillance system in Yukon. The original iteration of our ED-SyS was built using terms and case definitions informed by relevant literature, which did not capture the range of nuances inherent in our local data source. By reviewing and validating records on a case-by-case basis using our pragmatic approach tailored to the scarce resources available, we were able to identify additional terms and patterns of chart record-keeping that allowed us to make adjustments to the syndromic case definitions that improved the predictive ability of the ED-SyS. We found the CEDIS terms used in the CC field were especially useful for exploring false- positive cases flagged in our results. Having a standard terminology allowed us to group false positive cases from each syndrome by their CEDIS term and explore whether it was necessary to build additional exclusions into our syndromic case algorithms. This was not as feasible with either of the CN or DD fields, as they both contained free-text input, which proved much more variable than the CEDIS-codes and their standardized terms.

We were motivated to establish an ED-SyS to not only support surveillance activities during the mass-gathering events including the AWG, but also enhance Yukon’s ongoing surveillance infrastructure for detection and response capabilities. Our approach and ability to leverage multiple ED record fields resulted in final case definitions with moderate to high PPVs (62.5–94.1%). Moving forward, the acceptable balance between case detection and accuracy will need to depend on the risk posed by missing a case vs the burden of dealing with false positives. Under short term, high-risk situations including future AWG events, and for monitoring high profile conditions such as COVID-19, maximizing sensitivity will likely be prioritized. Where disease risk may be low, surveillance of syndromes with lower PPV may not warrant ongoing routine surveillance. For example, Yukon may consider discontinuing surveillance for the rash syndrome, given the low frequency of measles in the territory and lower PPV. Since validation, the ED- SyS was leveraged for example to support ongoing enhanced surveillance for respiratory, ILI, and COVID-19 during the influenza season and COVID-19 pandemic. Our ED-SyS did not detect any potential COVID-19 cases from our October 1, 2018 to April 30, 2019 study dataset likely due to the lack of travel related documentation in the ED record. Travel related screening questions for respiratory presentations to the ED were more widely implemented in early 2020. However, since mid-February 2020, the system has flagged a small number of ED visits that meet the COVID-19 case definition (data not included in present analysis), and while follow-up on these potential cases did not result in identifying anyone positive for COVID-19 infection, it does indicate our system is working as intended. Further validation of the COVID-19 algorithm may be warranted in a future analysis. As the dynamics of the COVID-19 outbreak progress, we will continue to adapt the algorithm as needed.

The US-CDC’s EARS-X tool required minimal resources, was easy to operate, and provided a quick means to identify aberrations for further investigation. While the ED-Tracker was recently launched and baseline data is limited at this time, other automated statistical algorithms may be explored that consider historical trends, seasonal, and day-of-week effects [4]. Future development of the ED-SyS will include expanding to the syndromic surveillance of other public health issues in Yukon, including opioids, cannabis, forest fires, and secondary health impacts of the COVID-19 pandemic.

## Conclusions/practice implications

Our study highlights the feasibility of implementing an automated ED-SyS and validating syndrome case definitions in a low-resourced/remote setting using simple tools, resources, and adapted gold standard methods. Our approach to developing an ED-SyS allowed Yukon to move away from “drop-in” paper-based methods to create a “low-tech” sustainable system that can be leveraged for other mass-gathering events, other emerging health issues of concern, and general ongoing surveillance. Our study also reinforces the importance and value of validating syndrome case definitions using local data. Importantly, our study provides a path forward for other lower-resourced rural/remote settings on how to develop and validate syndrome case definitions.

## Supplementary Information

**Additional file 1.**

## Data Availability

All programming code, including natural language processing algorithms that were developed for this manuscript have been made available publicly [57]. The data that support the findings of this study are available from Yukon Health and Social Services, but restrictions apply to the availability of these data, which were used under license for the current study and so are not publicly available. Data are however available from the authors upon reasonable request and with permission of the Yukon government.

## Abbreviations

AWG: Arctic winter games
ED: Emergency department
SyS: Syndromic surveillance system
CC: Chief complaint
DD: Discharge diagnosis
CN: Clinical (triage) notes
WHG: Whitehorse general hospital
CEDIS: Canadian emergency department information system
NACRS: National ambulatory care reporting system
ICD: International classification of disease
YCDC: Yukon center for disease control
OCMOH: Office of the chief medical officer of health
NC Detect: North Carolina disease event tracking and epidemiologic collection tool
ESSENCE-II: Electronic surveillance system for the early notification of community-based epidemics
COVID-19: Coronavirus-disease 2019
US-CDC: United States centers for disease control and prevention
GI: Gastrointestinal illness
ILI: Influenza-like illness
Neuro: Neurological illness
Resp: Respiratory illness
PPV: Positive predictive value
LUQ: Left upper quadrant
LLQ: Left lower quadrant
RLQ: Right lower quadrant
RUQ: Right upper quadrant
nyd: Not yet determined
